# Latent Profiles of Modifiable Biological Factors and Their Associations with Lifestyle Factors and Cardiovascular Disease Outcomes

**DOI:** 10.3390/jcm15093475

**Published:** 2026-05-01

**Authors:** Giedre Aukstakalniene, Renata Paukstaitiene, Abdonas Tamosiunas, Vaiva Lesauskaite, Dalia Luksiene

**Affiliations:** 1 Laboratory of Population Studies, Institute of Cardiology, Medical Academy, Lithuanian University of Health Sciences, Sukilėlių Ave. 15, 50103 Kaunas, Lithuania; giedre.aukstakalniene@lsmu.lt (G.A.); abdonas.tamosiunas@lsmu.lt (A.T.); 2 Department of Physics, Mathematics, and Biophysics, Medical Academy, Lithuanian University of Health Sciences, Eivenių St. 4, 50103 Kaunas, Lithuania; renata.paukstaitiene@lsmu.lt; 3 Laboratory of Molecular Cardiology, Institute of Cardiology, Medical Academy, Lithuanian University of Health Sciences, Sukilėlių Ave. 15, 50103 Kaunas, Lithuania; vaiva.lesauskaite@lsmu.lt; 4 Department of Environmental and Occupational Medicine, Public Health Faculty, Lithuanian University of Health Sciences, Tilžės St. 18, 47181 Kaunas, Lithuania

**Keywords:** latent profile analysis, biological risk factors, cardiovascular disease, lifestyle factors

## Abstract

**Background/Objectives**: There is growing interest among researchers in improved biological risk factor indices or combinations of indices, which have emerged as a major focus in evaluating the risk of cardiovascular disease (CVD). This study aimed to identify latent profiles based on the clustering of biological factors and to explore associations between lifestyle factors and CVD outcomes across the identified latent profiles. **Methods**: This epidemiological health survey was performed during 2023–2024. A random sample of Kaunas inhabitants aged 25–69 years, stratified by sex and age, was randomly selected from the Lithuanian population register. The 3426 individuals were screened. Latent profile analysis was performed using Latent Gold 6.1 and IBM SPSS Statistics 30. Multinomial logistic regression and multivariable binary logistic regression were used to evaluate the associations between biological risk factor profiles, lifestyle factors, and CVD outcome. **Results**: Three biological risk factor profiles were identified: low-risk profile (42.6%) was considered the healthiest, having the lowest levels of body mass index (BMI), fasting glucose, systolic blood pressure (SBP), and the highest level of high-density lipoprotein (HDL) cholesterol. Medium-risk profile (50.4%), having intermediate indicators of those factors. The high-risk profile (7.0%) was characterized by the lowest HDL cholesterol and the highest values of triglycerides, fasting glucose, SBP, and BMI. **Conclusions**: Compared to the low-risk profile, medium- and high-risk biological profiles were independently associated with higher odds of CVD, according to sociodemographic and lifestyle factors. The study suggests that integrating multiple biological risk factors rather than a single risk factor in clinical practice may enhance diagnostic accuracy.

## 1. Introduction

Modifiable biological risk factors—most notably elevated systolic blood pressure, abnormal lipid levels, increased blood glucose, and excess body weight—continue to rise worldwide and represent major threats to population health and life expectancy [1,2,3,4]. These conditions are not only preventable but also medically treatable. Understanding how these modifiable biological risk factors emerge, interact, and co-occur within the population is essential for clarifying pathways underlying cardiovascular disease (CVD) and for informing more effective prevention and management strategies. Despite notable progress in early detection and treatment over recent decades, CVD remains the leading cause of mortality in Lithuania. According to data from the Health Information Centre of the Institute of Hygiene, diseases of the circulatory system accounted for more than half (50.8%) of all deaths in 2025 [5].

Although biological indicators such as dyslipidemia, elevated blood pressure, and increased body mass index (BMI) are well-established contributors to CVD, diagnostic and predictive models that rely on individual markers—or even simple combinations of them—often exhibit limited sensitivity and specificity. A considerable proportion of individuals who eventually develop CVD do not display the conventional risk factors incorporated into current screening frameworks, resulting in many cases going undetected [6]. This diagnostic shortfall has intensified scientific interest in developing more informative biological indices and composite risk profiles that may better capture the complex and multifactorial nature of CVD susceptibility.

Latent profile analysis (LPA) is a statistical technique that uses categorical or continuous latent variables to identify hidden subgroups within a population based on a specified set of characteristics [7]. This method assigns individuals to different profiles probabilistically, reflecting the degree to which they fit a particular subgroup. A key strength of LPA is its ability to segment populations using multiple variables, making it highly effective for characterizing complex patterns, such as biological risk factor profiles in heterogeneous populations. Applying LPA to modifiable biological risk factors provides valuable insight into how these indicators tend to co-occur within individuals. Identifying patterns of joint occurrence can help clarify potential underlying mechanisms [8]. For example, if two risk factors consistently appear together more frequently than others, this may indicate that they share a common antecedent, such as a lifestyle-related behavioral influence. Moreover, when constellations of risk factors are disproportionately represented in specific population subgroups, it suggests that characteristics typical of those subgroups may contribute to the emergence of these combinations. Understanding these configurations is important because the clustering of modifiable biological risk factors may have synergistic effects that disproportionately increase the likelihood of negative CVD outcomes.

This study aimed to (1) identify latent profiles based on the clustering of biological factors among the 25–69-year-old population and (2) explore the associations between the lifestyle factors and CVD outcome with the identified latent profiles.

## 2. Materials and Methods

### 2.1. Study Population

The study “Chronic Diseases and Their Risk Factors in the Adult Population” was carried out in Kaunas, Lithuania, using standardized procedures commonly applied in international cardiovascular epidemiology research of the WHO MONICA study [9,10]. A stratified random sample of city residents aged 25–69 years was generated from the Lithuanian Population Register, with stratification by sex and 10-year age groups. To ensure adequate representation and account for expected participation rates of approximately 50–60%, 6000 individuals (3000 men and 3000 women) were invited to participate. Selected individuals received written invitations to attend a comprehensive health examination at the Hospital of the Lithuanian University of Health Sciences, Kaunas Clinics. The health examination began on 1 February 2020 but was interrupted on 15 March 2020 due to the COVID-19 pandemic. Fieldwork resumed in March 2023 once restrictions were lifted and continued until 21 June 2024. A total of 3426 participants completed the examination, corresponding to a response rate of 57.1%. The study protocol received approval from the Kaunas Regional Biomedical Research Ethics Committee (No. BE-2-49; 5 June 2018), and all participants provided written informed consent. All invited individuals were eligible for participation; no exclusion criteria were applied. After removing 15 individuals with missing essential data (e.g., unsuccessful or refused blood sampling, incomplete questionnaires), 3411 participants were included in the final analysis.

### 2.2. Variables Determined Using the Questionnaire

During the health examination, trained interviewers gathered sociodemographic and health-related data using a structured questionnaire, which included items on age, sex, and educational level, self-rated health, and smoking habits. Evaluation and classification of sociodemographic factors, self-rated health, and smoking habits are presented in detail in our previous publication [11].

Physical activity was assessed as the mean number of hours per week that participants spent on leisure-time activities during summer–autumn and winter–spring, including walking, gardening, house maintenance, and other physical activities. Participants were then divided into five equal groups (quintiles) based on their mean weekly time spent on physical activity. The upper cut-off values for the quintiles were as follows: 1st quintile: 3.42 h/week (mean 1.79; SD 1.10); 2nd quintile: 6.00 h/week (mean 4.80; SD 0.81); 3rd quintile: 8.50 h/week (mean 7.40; SD 0.71); 4th quintile: 12.50 h/week (mean 10.53; SD 1.14); 5th quintile: 42.00 h/week (mean 19.10; SD 6.82)

Dietary habits were assessed using a semi-quantitative food-frequency questionnaire covering 30 commonly consumed food groups. Participants reported how often they consumed items such as cereals and porridges, pasta, legumes, whole-grain bread, potatoes (boiled or fried), dairy products (e.g., cheese, curd, sour cream, milk), various types of meat, smoked meat products, fish, eggs, fresh vegetables, boiled or canned vegetables, fresh fruits and berries, sweets, cakes, fast-food items, salty snacks, and sugar-sweetened beverages. Seasonal variation was captured by asking separately about consumption of fresh vegetables, fruits, and berries in summer–autumn and in winter–spring. For each food group, respondents selected one of six frequency categories: 1. rarely or never; 2. 1–3 times per month; 3. once per week; 4. several times per week; 5. daily, and several times per day. Higher values indicated more frequent consumption. To identify underlying dietary patterns, exploratory factor analysis was applied to the frequency data. Details of the analytical approach have been described previously [11]. The analysis yielded six major dietary patterns: fast-food consumption; meat products and potatoes; fresh vegetables, fruits, and fish; dairy products; sweets; porridges, cereals, and pasta. For subsequent analyses, each factor score was dichotomized to distinguish participants with above-average consumption of a given pattern (coded as 1) from those with lower-than-average consumption (coded as 0).

### 2.3. Measurements

*Anthropometry.* Body weight was measured using a calibrated medical scale with participants wearing light clothing and no shoes. Measurements were recorded to the nearest 0.1 kg. Standing height was assessed without shoes using a stadiometer, accurate to 1 cm. BMI was calculated as weight in kilograms divided by height in meters squared. Participants were categorized as follows: underweight (BMI < 18.5 kg/m^2^), normal weight (18.5–24.99 kg/m^2^), overweight (25.0–29.99 kg/m^2^), and obese (≥30.0 kg/m^2^).

*Blood pressure.* Systolic and diastolic blood pressure were measured twice using an automated oscillometric device (Omron M5-1, Medaval, Cork City, Republic of Ireland) after the participant had been seated at rest for at least five minutes. The mean of the two readings was used in the analysis.

*Biochemical analyses.* After an overnight fast of at least eight hours, venous blood samples were collected into serum tubes, ensuring samples were free of haemolysis. For each biochemical parameter, 200 µL of serum was analyzed. Triglycerides, high-density lipoprotein (HDL) cholesterol, and glucose were measured using enzymatic colorimetric methods on a Selectra PRO XS analyzer (EliTech Group, Rotterdam, The Netherlands). Reagents for HDL cholesterol and triglycerides were obtained from EliTech Group B.V. Absorbance was read at 500 nm. Calibration followed manufacturer recommendations, using Randox cholesterol calibrators, HDL-cholesterol calibrators, and Clinical Chemistry Calibration Serum Level 3 (Randox, Crumlin, UK). Internal quality control was performed daily using Randox Lipid Control Levels 1–3, and results were compared with expected values to ensure analytical precision. Plasma glucose was determined using the GLUCOSE OXIDASE-PAP/End Point Enzymatic PAP method on the same analyzer.

The diagnostic criteria for metabolic syndrome are based on the Third Report of the National Cholesterol Education Program Adult Treatment Panel III (NCEP-ATP III) criteria [12].

### 2.4. Identification of CVD

CVD was defined as the presence of ischemic heart disease (IHD) and/or a history of stroke. Several sources of information were used to identify these conditions.

Participants were first asked whether they had ever experienced a myocardial infarction (“Have you ever had a myocardial infarction?”; response options: yes/no). Those who reported a previous event were asked to indicate the year and the hospital where they received treatment. In addition, a resting electrocardiogram (ECG) was recorded for every participant and coded according to the Minnesota classification system.

IHD at the time of examination was determined using the following hierarchical criteria: evidence of previous myocardial infarction and/or major ischemic ECG abnormalities (Minnesota codes 1–1, 1–2) [13]; symptoms of exertional angina pectoris identified using the Rose questionnaire [14]; other ischemic ECG changes based on Minnesota codes 1–3, 4–1, 4–2, 4–3, 5–1, 5–2, 5–3, 6–1, 6–2, 7–1, and 8–3 [13].

A history of stroke was assessed with the question: “Have you ever had a stroke?” (yes/no). Participants who answered “yes” were asked to specify the year and hospital of treatment.

To assess medication use, respondents were asked: “Are you currently taking any medication for cholesterol reduction?” “Are you currently taking any medication for blood glucose control?” “Are you currently taking any medication for blood pressure control?” Possible answers: Yes/No.

### 2.5. Statistical Analysis

All statistical procedures were performed using IBM SPSS Statistics, Version 30.0 (IBM Corp., Armonk, NY, USA). Descriptive characteristics of the study population were presented as mean ± standard deviation or as median with minimum and maximum values for continuous variables, depending on distributional properties. Categorical variables were presented as counts and percentages. The distribution of quantitative variables was evaluated using the Kolmogorov–Smirnov test. Comparisons across three groups were conducted using the Kruskal–Wallis test, followed by Bonferroni-adjusted post hoc analyses when appropriate. Results for non-normally distributed variables are reported as medians, ranges, and interquartile ranges (IQR). Differences in categorical variables were examined using the chi-square test for homogeneity, with Bonferroni-corrected Z-tests applied for pairwise comparisons. A *p*-value below 0.05 was considered statistically significant.

Latent profile analysis was used to identify clusters of biological risk factors. The analysis was performed using the Latent Gold 6.1 software. Continuous variables (systolic arterial blood pressure, fasting glucose, triglycerides, HDL cholesterol, total cholesterol, and BMI) were used as indicators, and categorical variables (use of medications for control of high blood pressure, cholesterol, and glucose levels) as active covariates. The number of latent profiles selected for the analysis was determined based on a combination of fit indices (Akaike Information Criterion (AIC), Bayesian Information Criterion (BIC), Sample-size Adjusted Bayesian Information Criterion (SABIC), and Consistent Akaike Information Criterion (CAIC)), log-likelihood values, entropy, and classification error rates. Lower values of these indices indicated better model fit, and entropy above 0.7, together with classification error below 0.1, suggested good profile separation.

Multinomial logistic regression was used for the analysis of biological risk factor profiles. The low-risk profile chosen as the reference category allowed us to assess how demographic, clinical, and lifestyle factors increase or decrease the odds of entering higher-risk profiles. Adjusted odds ratios (aOR) and their 95% confidence intervals were calculated to assess the independent influence of each variable on the odds of being in a higher-risk profile.

Multivariable binary logistic regression was used to evaluate the association of biological risk factor profiles, lifestyle factors, and CVD outcome. The dependent variable was CVD. Independent variables were the three biological risk factor profiles, lifestyle habits (smoking status, physical activity status, nutrition habits), and 3 groups of self-rated health, sex, age groups, and education status. The aOR and their 95% confidence intervals were calculated to assess the independent influence of each variable on the odds of CVD.

## 3. Results

The characteristics of participants are presented in Table 1. Data of 3411 participants (1515 males and 1896 females) were available for statistical analysis.

The participants’ average age was 49.1 (SD = 11.2) years, and the median age was 50 years. The distribution according to education level revealed a high prevalence of responders with a university degree (49.8%), 31.1% of responders had completed secondary and lower education, and 19.1% had a college degree. More than half of responders (58.9%) were defined as never smokers, and 19.6% were current smokers. Self-rated health as good indicated more than half of the respondents (61.0%) (Table 1).

Table 2 presents the summary of latent profile model identification and model fit indicators. Models with 2–4 profiles were estimated.

The three-profile model was chosen for further analysis because it showed the best classification and model fit results. Its fit indices (AIC = 77,727.29; BIC = 77,997.22; SABIC = 77,857.41; CAIC = 78,041.22) and log-likelihood (LL = −38,819.64) were clearly lower compared with the two-profile model, but slightly higher compared with the four-profile model. In contrast, entropy (0.74) and classification error (0.096) were slightly lower compared with the two-profile model, and clearly higher compared with the four-profile model (Table 2). While the 4-profile model showed better fit indices, its classification results were worse than those in the 3-profile model: moving from three to four profiles decreased the entropy R^2^ by 8% (from 0.74 to 0.68), and the classification error increased by 60% (from 0.096 to 0.16). Furthermore, the third class in the 3-profile model clearly represents a high-risk subgroup (significantly higher systolic arterial blood pressure, fasting glucose, and other biological indicators), while the 4-profile model, however, did not distinguish between fundamentally different subclasses; instead, it divided one of the profiles in the three-profile model into two quantitatively similar subgroups with only minor differences in biological indicators. Also, the smallest subclass in the 4-profile model, compared to the smallest subclass in the 3-profile model, decreased to 6.5% from 7.8%. This further confirmed that the three-profile solution is the most interpretable and theoretically meaningful option. Although the four-profile solution resulted in slightly improved fit indices, further examination indicated limited biological interpretability. The four-profile model primarily reflected a redistribution of individuals from the three-profile structure and yielded an additional profile that did not differ meaningfully from other profiles across most biological indicators in pairwise comparisons. Moreover, the four-profile solution did not provide greater differentiation with CVD outcomes than the three-profile solution. Taken together, the additional profile did not represent a distinct biological risk phenotype, but increased model complexity without improving interpretability. Therefore, the three-profile solution was retained as the most parsimonious and biologically meaningful representation.

Table 3 presents the size of each profile and the distributions of lipids, systolic arterial blood pressure, BMI, and the use of medications to lower cholesterol, glucose levels, and blood pressure across the profiles. The profiles were categorized based on the relative magnitudes of the lipid levels, systolic blood pressure, BMI, and use of medication as follows: low-risk profile, the highest HDL cholesterol and the lowest triglycerides, fasting glucose levels, systolic blood pressure, and BMI. The proportions of responders using medication to lower blood pressure, cholesterol, and glucose levels were also lowest in this profile. The medium-risk profile is characterized by the highest level of total cholesterol and intermediate indicators of other variables, compared to the other two profiles. High-risk profile, the lowest HDL cholesterol, and the highest systolic blood pressure, BMI, other lipid levels (except for total cholesterol), and the highest proportions of responders using medication (Table 3).

Table S1 presents the distribution of sociodemographic and lifestyle factors according to biological factor profiles. Multinomial logistic regression was conducted to examine the association between sociodemographic and lifestyle factors and membership in biological risk factor profiles, using the low-risk profile as the reference category (Table 4).

Age showed a significant association with profile membership. Compared with adults aged 25–34 years, all older age groups had significantly higher odds of belonging to the medium or high-risk profiles. For the medium-risk profile, the aORs increased from 1.66 (95% CI: 1.27–2.18) in the 35–44 age group to 5.99 (95% CI: 4.48–7.99) among those aged 55+. A similar pattern was observed for the high-risk profile, where the oldest age group (55+) had nearly a sevenfold increase in odds (aOR = 6.96; 95% CI: 3.82–12.67). All associations for age were statistically significant, except for the 35–44 age group in the high-risk profile. Sex was also significantly associated with profile membership. Compared with males, females had substantially lower odds of belonging to either the medium-risk (aOR = 0.37; 95% CI: 0.31–0.44) or high-risk profile (aOR = 0.25; 95% CI: 0.18–0.34). These findings indicate that males were more likely to be in higher-risk biological profiles.

Education level was associated with biological risk profile membership (Table 4). Using secondary or lower education level as the reference, university education was associated with reduced odds of being in both the medium-risk (aOR = 0.82; 95% CI: 0.68–0.99) and high-risk profiles (aOR = 0.63; 95% CI: 0.45–0.89). College education did not show significant associations with any of the profiles. Smoking status and physical activity in leisure time were not significantly associated with membership in the medium and higher-risk profiles. Despite that, the lower levels of leisure time physical activity (1st quintile) were associated with a higher prevalence of medium and high-risk profile membership in descriptive analyses; however, after adjustment, the association was not statistically significant. Compared to the lower-risk profile, responders in the medium-risk and higher-risk profiles were significantly more frequently consuming meat products and potatoes (aOR = 1.41; 95% CI: 1.20–1.65 and aOR = 1.48; 95% CI: 1.07–2.03, respectively), and less frequently consuming fresh vegetables, fruits, and fish (aOR = 0.83; 95% CI: 0.71–0.97 and aOR = 0.67; 95% CI: 0.49–0.90, respectively) and less frequently consuming sweets (aOR = 0.75; 95% CI: 0.65–0.88 and aOR = 0.45; 95% CI: 0.33–0.61, respectively) and cereals/porridge (aOR = 0.77; 95% CI: 0.66–0.89 and aOR = 0.70; 95% CI: 0.52–0.94, respectively). Responders who reported self-rated health as average and poor had a higher odds of being in the medium-risk and higher-risk profiles compared to the reference low-risk profile (aOR = 1.58; 95% CI: 1.34–1.87 and aOR = 2.41; 95% CI: 1.77–3.28, respectively).

Table S2 presents the distribution of modifiable biological risk factor profiles, self-rated health, and lifestyle factors according to CVD outcome. Table 5 presents the associations between modifiable biological risk factor profiles, lifestyle factors, self-rated health, and CVD outcome in the 25–69-year-old population. Multivariable binary logistic regression analysis, including sex, age, and education, results show that responders from medium-risk and high-risk profiles had higher odds of CVD compared to responders from low-risk profiles (aOR = 1.59; 95% CI: 1.23–2.06 and aOR = 2.52; 95% CI: 1.70–3.75, respectively). Responders who assessed their health as average and poor compared to responders who assessed their health as good had significantly higher odds of CVD (aOR = 1.41; 95% CI: 1.13–1.77). From all analysed lifestyle factors (smoking, physical activity, dietary habits), only more frequent consumption of meat products and potatoes was positively associated with CVD (aOR = 1.32, 95% CI: 1.04–1.66) among the population aged 25–69 years (Table 5). We compared two different models for evaluation of the association of CVD outcome: first, using modifiable biological risk factor profiles, and second, using metabolic syndrome, but with the same other variables in both models (self-rated health and lifestyle factors). Across all evaluated metrics, the model, with biological risk factor profiles, compared with the model including metabolic syndrome, demonstrated consistently superior performance. First, the model, with biological risk factor profiles, showed a lower −2 Log Likelihood (2308.879 vs. 2326.072) and a larger model chi-square (141.337 vs. 128.169), indicating improved explanatory power. The difference in model fit was statistically significant (Δ(−2LL) = 17.193, df = 1, *p* < 0.001), demonstrating that the model with biological risk factor profiles provides additional information compared to the model with the metabolic syndrome indicator. Pseudo R^2^ measures further corroborated this improvement (Nagelkerke R^2^ = 0.079 vs. 0.072). Importantly, information-theoretic indices—AIC (2346.879 vs. 2362.072) and BIC (2463.254 vs. 2472.322)—also favored the model, with biological risk factor profiles, even after penalizing the slightly increased model complexity.

## 4. Discussion

Using latent profile modeling, we identified distinct biological risk factor profiles and demonstrated their associations with sociodemographic characteristics and CVD risk in a population-based study of adults aged 25–69 years. Our study identified three biological risk factor profiles. The medium-risk profile was the most prevalent (50.4%) and was characterized by the highest total cholesterol levels alongside intermediate values of systolic blood pressure, lipid parameters, BMI, and fasting glucose. In contrast, the high-risk profile (7.0%), although least prevalent, represented a metabolically adverse phenotype, combining low HDL cholesterol with elevated triglycerides, fasting glucose, blood pressure, and BMI. The low-risk profile (42.6%) reflected a more favorable cardiometabolic state, with the lowest levels of BMI, fasting glucose, and blood pressure, and the highest HDL cholesterol levels. The prevalence of profiles or classes reported in epidemiological studies varies with the number of profiles and factors included in the latent profile or class analysis. The predominance of a medium-risk group in our study is consistent with findings from other populations, suggesting that a substantial proportion of adults may be in a transitional cardiometabolic state rather than at the extremes of risk. Similar patterns have been observed in studies from South China and Iran, where intermediate or lifestyle-related risk profiles were also the most prevalent [15,16]. This consistency across diverse populations may indicate that clustering of moderately elevated levels of factors could represent an early stage of cardiometabolic dysregulation, potentially preceding the development of more severe high-risk phenotypes. The results of the cross-sectional study in South China among adults aged 20–80 years also showed, as in our study, that the class with “moderate risk perception” was the most prevalent (46.8%) [15]. In the cross-sectional study of the Kharameh cohort (Iran), analyzing the patterns of type 2 diabetes risk factors, three classes of risk factors were determined, and the most prevalent (61%) was the so-called “lifestyle-risk” class [16]. However, differences in the number and composition of identified profiles across studies highlight the methodological sensitivity of latent profile analysis to the selection of included variables and population characteristics. For example, a study among cardiology inpatients in China identified four lipid-based profiles, with the healthiest profile being the most common [8], which may reflect clinical selection and a narrower focus on lipid parameters. In contrast, our population-based approach incorporating multiple biological risk factors provides a more comprehensive representation of cardiometabolic factor clustering. Taken together, these findings suggest that while the general pattern of factors clustering is reproducible across settings, the specific profile structure and prevalence may vary depending on both methodological choices and population context. Importantly, the identification of a substantial medium-risk group highlights the need for early, population-level prevention strategies directed toward individuals who may not yet be classified as high-risk but who already display unfavorable combinations of risk factors.

The results of many scientific studies show that these biological risk factors are significantly interconnected. This interrelated pattern is consistent with the clustering of risk factors observed in our identified profiles, suggesting that these variables do not act independently but reflect underlying shared pathophysiological processes. The findings indicate that associations between BMI and both blood pressure and lipid variables are largely independent of plasma glucose levels [17]. Higher fasting glucose levels are linked with poor insulin sensitivity and pre-diabetes [18]. Lipid abnormalities—most notably elevated triglyceride concentrations and reduced HDL cholesterol—are associated with higher fasting glucose levels in the general population, indicating that disturbances in lipid metabolism frequently accompany impairments in glucose regulation [18]. The results from the China Health and Retirement Longitudinal Study (CHARLS) showed that overweight and obesity significantly increased the odds of hypertension in both men and women, whereas elevated triglyceride levels were associated with hypertension only in women [19]. Elevated blood pressure shares common pathogenic pathways with dyslipidemia and glucose abnormalities, often due to endothelial dysfunction (damage to blood vessel lining) and increased vascular resistance caused by metabolic inflammation and insulin resistance. These mechanisms may explain the co-occurrence of elevated blood pressure, adverse lipid profiles, and increased BMI within the high-risk profile identified in our study. The research data from the Norwegian population (*n* = 109,796) aged 40–45 years, show that controlling triglycerides and blood pressure in middle-aged individuals should be targeted to prevent later onset of type 2 diabetes mellitus [20]. Jin et al. [21] conducted a prospective cohort study that included 2659 adults aged 20–74 years to investigate the temporal relationships between blood glucose, lipid parameters, and BMI and their effects on atherosclerosis risk. The findings of this study, the Harbin Cohort Study on Diet, Nutrition and Chronic Non-communicable Diseases, suggest that metabolic disturbances in glucose and lipid metabolism may precede and contribute to increases in BMI, which in turn elevate atherosclerosis risk. This temporal sequence supports the interpretation that the profiles identified in our study may represent different stages of cardiometabolic progression rather than static risk categories, highlighting the importance of early and integrated cardiometabolic risk prevention strategies.

The next part of this study was designed to assess the associations between the sociodemographic and lifestyle factors, identified latent profiles, and CVD outcome.

Age showed a strong, graded association with membership in both medium- and high-risk profiles. Participants aged 55 years and older had approximately six- to seven-fold higher odds of belonging to higher-risk profiles compared with those aged 25–34 years. This graded relationship is consistent with extensive epidemiological evidence demonstrating age-related accumulation of cardiometabolic risk factors, including hypertension, dyslipidemia, impaired glucose metabolism, and increased adiposity [22]. Ageing is associated with progressive vascular stiffening, endothelial dysfunction, and metabolic dysregulation, which may contribute to the transition from lower- to higher-risk profiles observed in our study, explaining the clustering of adverse biological characteristics in older adults [23].

Sex differences were also pronounced. Females had substantially lower odds of belonging to medium- and high-risk profiles compared with males. This aligns with prior evidence indicating that men tend to develop cardiometabolic risk factors earlier in life, whereas premenopausal women are partially protected by hormonal and metabolic factors [24]. This sex-specific pattern may indicate that the distribution of latent risk profiles is partly shaped by hormonal regulation and differences in fat distribution and metabolism. However, this advantage may diminish with age, particularly after menopause, potentially leading to a convergence of risk profiles between men and women in later life, suggesting the importance of sex-specific prevention strategies across the life course.

Higher educational attainment was inversely associated with membership in the high-risk profile, particularly among individuals with university education. These findings are consistent with the well-documented social gradient in cardiovascular health, whereby individuals with higher socioeconomic status tend to exhibit more favorable risk profiles and lower CVD incidence [25]. Education can shape health literacy, access to preventive services, dietary quality, occupational exposures, and long-term health behaviors, thereby influencing both individual risk factors and their clustering into more or less favorable cardiometabolic profiles.

Self-rated health was strongly associated with both biological risk clusters and CVD. Individuals reporting average or poor health had significantly higher odds of belonging to higher-risk profiles and of having CVD. Self-rated health is a robust predictor of morbidity and mortality and may integrate subclinical symptoms, psychosocial stress, and undiagnosed conditions into a single subjective measure [26], which may explain its strong alignment with objectively measured risk profiles in our study.

Smoking status and leisure-time physical activity were not independently associated with profile membership after adjustment, despite descriptive differences in physical activity levels. This finding is somewhat unexpected given the well-established role of these behaviors in the development of cardiometabolic risk. Smoking and physical activity often associated with age, sex, and education. For this reason, smoking and physical activity lost their statistical association with latent profiles after adjustment because their apparent effects were actually explained by other, more proximal or more powerful variables in the model (e.g., age, sex, education). Thus, after adjustment for confounding variables, the independent contribution of smoking or physical activity is no longer evident. Moreover, beyond the scope of our study, additional unmeasured variables—such as genetic predispositions related to cardiometabolic risk—may also influence biological factor profiles [27]. Also, smoking and physical activity were based on self-reported information, which may introduce misclassification or reporting bias. Alternatively, these behaviors may influence risk indirectly through long-term biological pathways that are not fully captured in cross-sectional analyses or may already be reflected in the biological variables included in the profiles. Previous studies have consistently demonstrated associations between smoking and physical inactivity and cardiometabolic risk [28,29], suggesting that their impact may operate indirectly through long-term biological changes not fully captured in cross-sectional analyses.

Dietary patterns, however, showed more consistent associations. Membership in medium- and high-risk profiles was positively associated with more frequent consumption of meat products and potatoes and inversely associated with intake of fresh vegetables, fruits, fish, cereals/porridge, and sweets. The positive association with meat consumption is consistent with evidence linking high intake of red and processed meats to increased cardiometabolic and CVD risk, potentially mediated by saturated fat, sodium, and pro-inflammatory compounds [30]. Conversely, higher consumption of vegetables, fruits, whole grains, and fish is a hallmark of cardioprotective dietary patterns, such as the Mediterranean diet, which has been associated with improved lipid profiles, glycemic control, and reduced inflammation [31,32]. Our results demonstrated that among lifestyle factors, only frequent consumption of meat products and potatoes remained independently associated with CVD in multivariable models. This suggests that specific dietary components may exert a direct influence on cardiovascular risk beyond their contribution to biological risk factor clustering, highlighting their potential role as independent targets for prevention. Interestingly, lower consumption of sweets was associated with higher-risk profiles. This counterintuitive finding may reflect reverse causation, whereby individuals with diagnosed metabolic disturbances reduce their intake of sugary foods following medical advice. One of the main recommendations for people diagnosed with type 2 diabetes is to limit their intake of simple carbohydrates (sweets). Lowering intake of sugary beverages, white bread, and pastries to improve insulin sensitivity and glycemic control [33]. Despite that, as indicated in those recommendations, some individuals with overweight or obesity could often report lower sugar or sweets intake than lean individuals. For these reasons, findings related to carbohydrate intake—particularly the consumption of sweets—should be interpreted with caution due to the potential for reporting bias [33].

Most importantly, both medium- and high-risk biological profiles were independently associated with higher odds of CVD, even after adjustment for sex, age, and education. The stepwise increase in CVD odds across profiles supports the concept that cardiometabolic factors act synergistically rather than independently. Clustering of hypertension, dyslipidemia, hyperglycemia, and excess adiposity likely reflects shared pathophysiological mechanisms—such as insulin resistance, chronic inflammation, and endothelial dysfunction—that accelerate atherosclerotic processes [8,34]. These findings reinforce the validity of the identified profiles as clinically meaningful representations of cardiometabolic risk. The well-established metabolic syndrome cluster—characterized by elevated arterial blood pressure, dyslipidemia, hyperglycemia, and increased waist circumference—is widely used in epidemiological research to evaluate CVD outcomes. In this study, we compared two distinct modeling approaches for assessing the association with CVD outcomes: first, a model incorporating modifiable biological risk factor profiles, and second, a model based on metabolic syndrome, while holding constant the same additional covariates (self-rated health and lifestyle factors) in both models. Across all evaluated metrics, the model, with biological risk factor profiles, compared with the model including metabolic syndrome, demonstrated consistently superior performance.

The novel contribution of our study: first, that we used LPA, which assigns individuals to profiles probabilistically, allowing a more nuanced representation of how people fit into complex cardiometabolic subgroups in a large population-based sample (*n* = 3411) with a wide age range (25–69 years). Second, we performed the direct comparison of LPA-derived profiles with the widely used metabolic syndrome construct for association with CVD risk. To our knowledge, few studies have systematically compared LPA-based risk profiles with metabolic syndrome while holding all other covariates constant. Taken together, these results provide consistent evidence that using modifiable biological risk factor profiles offers a more informative and statistically robust representation of CVD outcome than the binary metabolic syndrome variable.

Therefore, this study suggests that integrating multiple biological risk factors rather than a single risk factor in clinical practice may enhance diagnostic accuracy. This approach may provide added value compared with traditional single-factor risk assessment models, particularly in identifying individuals with combined moderate abnormalities who may otherwise remain undetected. For instance, individuals with a high-risk profile, characterized by the lowest HDL cholesterol and the highest values of triglycerides, fasting glucose, systolic blood pressure, and BMI, had a higher CVD risk compared to the group with a low-risk profile, having the lowest levels of BMI, fasting glucose, systolic blood pressure, and the highest level of HDL cholesterol. This highlights the importance of a comprehensive assessment of biological risk factors for patient evaluation and risk stratification, and underscores the potential hazards associated with elevated triglycerides, fasting glucose, systolic blood pressure, BMI, and reduced HDL levels. Such patients may require closer monitoring and more vigilant care in clinical practice. From a public health perspective, these findings emphasize the importance of early and integrated prevention strategies targeting clusters of modifiable risk factors rather than isolated behaviors or biomarkers. Taken together, these findings underscore the importance of integrated prevention strategies targeting profiles of modifiable biological risk factors, particularly among older adults, men, and individuals with lower educational attainment. Public health interventions should prioritize comprehensive risk assessment and multifactorial management, consistent with recommendations from international cardiovascular prevention guidelines. Future longitudinal studies are warranted to clarify causal pathways, assess temporal relationships, and evaluate whether targeted modification of high-risk clusters leads to meaningful reductions in CVD incidence.

### Study Strengths and Limitations

This study possesses several notable strengths. First, the innovative application of latent profile analysis to stratify biological risk factors enabled the identification of previously unrecognized risk profiles. These profiles enhance understanding of distinct risk factor subtypes within the population. By simultaneously considering multiple variables, LPA classifies individuals into homogeneous subgroups, making it particularly well-suited for delineating biological risk patterns. Second, the study demonstrated a strong association between the identified risk profiles and CVD outcomes. This finding is important for identifying populations at elevated risk and may inform more targeted prevention and intervention strategies. Third, we compared two distinct modeling approaches to evaluate the association with CVD outcomes: across all evaluated metrics, the model with biological risk factor profiles, compared with the model including metabolic syndrome, demonstrated consistently superior performance.

This study has several limitations that should be acknowledged. First, its cross-sectional design restricts the ability to draw causal inferences or to examine how biological risk factors interact and evolve over time. Because exposure and outcome were measured simultaneously, the possibility of reverse causality between risk factors and prevalent CVD cannot be excluded. Second, some variables—such as lifestyle behaviors (nutrition habits, physical activity, and smoking), self-rated health, and using self-reported history of myocardial infarction and/or stroke (for evaluation of CVD outcome)—were based on self-reported information, which may introduce misclassification or reporting bias. These limitations underscore the need for future longitudinal research to more rigorously elucidate causal pathways and to determine how the identified biological risk profiles relate to cardiovascular and other non-communicable disease outcomes.

## 5. Conclusions

Our study identified three profiles of biological factors and demonstrated their associations with sociodemographic characteristics and CVD risk in a population-based study of adults aged 25–69 years. Thus, the study suggests that cardiometabolic risk arises from complex, multi-organ biological interactions, underscoring the need to consider integrated profiles of biological risk factors—and their management—when assessing CVD risk, including regulation of glucose and lipid metabolism, control of blood pressure, and maintenance of a healthy BMI. Overall, this study suggests that integrating multiple biological risk factors rather than a single risk factor in clinical practice may enhance diagnostic accuracy.

## Figures and Tables

**Table 1:** Characteristics of participants of the Kaunas health survey (2023–2024).

Variables	Value
Number of responders, N	3411
Age, years, mean (SD)/median(min–max)	49.1 (11.2)/50 (25–69)
Age groups *n* (%)	
25–34	432 (12.6)
35–44	827 (24.1)
45–54	853 (24.9)
55+	1314 (38.4)
Sex	
Male, *n* (%)	1515 (44.4)
Female	1896 (55.6)
Education, *n* (%)	
Secondary and lower	1060 (31.1)
College	651 (19.1)
University	1696 (49.8)
Systolic arterial blood pressure, mm/Hg, mean (SD)/median (min–max)	132.8 (18.5)/131.5 (86–221)
Total cholesterol, mmol/L, mean (SD)/median (min–max)	5.55 (1.24)/5.44 (1.78–14.06)
HDL cholesterol, mmol/L, mean (SD)/median (min–max)	1.31 (0.46)/1.25 (0.1–4.19)
Triglycerides, mmol/L, mean (SD)/median (min–max)	1.42 (0.98)/1.18 (0.13–16.5)
Fasting glucose, mmol/L, mean (SD)/median (min–max)	5.55 (1.07)/5.4 (1.6–20.5)
Body mass index, kg/m^2^, mean (SD)/median (min–max)	27.3 (5.3)/26.7 (15.2–57.3)
Smoking habits, *n* (%)	
Never	2009 (58.9)
Former	734 (21.5)
Current smokers	665 (19.6)
Physical activity in leisure time, hours/week, mean (SD)/median (min–max)	
Physically inactive (1st quintile)	1.79 (1.1)/2.0 (0–3.42)
Physically active (2nd–5th quintile)	10.3 (6.4)/8.5 (3.5–42)
Nutrition habits, *n* (%)	
More frequent * fast-food consumption	1502 (44.1)
More frequent consumption of meat products and potatoes	1837 (54.0)
More frequent fresh vegetables, fruit, and fish consumption	1796 (52.8)
More frequent dairy product consumption	1799 (52.9)
More frequent sweets consumption	1773 (52.1)
More frequent porridge, cereals, and pasta consumption	1647 (48.4)
Uses medication, *n* (%)	
To lower blood pressure	895 (26.2)
To lower the cholesterol level	484 (14.2)
To lower the glucose level	122 (3.6)
Self-rated health, *n* (%)	
Good	2080 (61.0)
Average	1209 (35.4)
Poor	122 (3.6)
Metabolic syndrome, *n* (%)	1086 (31.8)
CVD, *n* (%)	404 (11.8)

SD—standard deviation, HDL—high-density lipoprotein, CVD—cardiovascular diseases. More frequent *—the proportions of more-frequent-than-average consumption of food items within each factor-derived food group.

**Table 2:** Summary of latent profile model identification and model fit indicators.

Class	AIC	AIC3	BIC	SABIC	CAIC	LL	Npar	Entropy	Class Err.
2	79,747.11	79,775.11	79,918.88	79,829.91	79,946.88	−39,845.55	28	0.75	0.06
3	77,727.29	77,771.29	77,997.22	77,857.41	78,041.22	−38,819.64	44	0.74	0.096
4	77,041.71	77,101.71	77,409.80	77,219.15	77,469.80	−38,460.86	60	0.68	0.16

AIC—Akaike Information Criterion based on the log-likelihood, AIC3—Akaike Information Criterion with penalty factor 3, BIC—Bayesian Information Criterion based on the log-likelihood, SABIC—Sample-size Adjusted Bayesian Information Criterion, CAIC—Consistent Akaike Information Criterion, LL—Log-likelihood value of the model, Npar—Number of free parameters estimated in the model, Entropy—Entropy-based measure of class separation (model-based R^2^), Class Err.—Classification error rate.

**Table 3:** Distribution of biological indicators and medicine use by latent profile membership.

		Profiles		
Variables	Low-Risk	Medium-Risk	High-Risk	H; *p* Value
Number of responders, *n* (%)	1452 (42.6)	1721 (50.4)	238 (7.0)	
Systolic arterial blood pressure, mm/Hg, median (min–max; IQR)	120.5 (86–159; 16)	139 (86–215; 21) ^a^	147.5 (104–221; 26) ^a,b^	1110.1; <0.001
Fasting glucose, mmol/L, median (min–max; IQR)	5.11 (2.95–6.51; 0.62)	5.62 (3.5–7.63; 0.82) ^a^	7.00 (1.60–20.50; 2.55) ^a,b^	910.1; <0.001
Triglycerides, mmol/L, median (min–max; IQR)	0.9 (0.13–2.01; 0.42)	1.51 (0.23–4.02; 0.94) ^a^	2.37 (0.34–16.5; 0.85) ^a,b^	1116.9; <0.001
HDL cholesterol, mmol/L, median (min–max; IQR)	1.43 (0.4–4.19; 0.61)	1.14 (0.1–3.0; 0.52) ^a^	0.97 (0.24- 2.51; 0.49) ^a,b^	48.7; <0.001
Total cholesterol, mmol/L, median (min–max; IQR)	5.3 (2.03–10.33; 1.51)	5.56 (2.08–10.15; 1.63) ^a^	5.27 (1.78–14.06; 2.2) ^c^	31.9; <0.001
Body mass index, kg/m^2^, median (min–max; IQR)	23.72 (15.2–32.5; 4.3)	28.93 (15.9–45.0; 5.7) ^a^	32.48 (20.0–57.3; 8.6) ^a,b^	1361.1; <0.001
Uses medication, *n* (%)				χ^2^; *p* value
To lower blood pressure	36 (2.5)	693 (40.3) ^a^	166 (69.7) ^a,b^	831.3; <0.001
To lower the cholesterol level	72 (5.0)	334 (19.4) ^a^	78 (32.8) ^a,b^	207.6; <0.001
To lower the glucose level	4 (0.3)	12 (0.7)	106 (44.5) ^a,b^	1245.1; <0.001

H—The differences among the three groups were assessed by the Kruskal–Wallis test and post hoc test with Bonferroni correction. Results were denoted as median (minimum–maximum) and interquartile range (IQR). A Chi-squared (χ^2^) test for homogeneity and a post hoc test (Z-test) with Bonferroni correction were used to determine differences in categorical variables. ^a^—*p* < 0.001 as compared to the Low-risk profile, ^b^—*p* < 0.001 as compared to the Medium-risk profile, ^c^—*p* < 0.05 as compared to the Medium-risk profile.

**Table 4:** Association of biological risk factor profiles with sociodemographic and lifestyle factors.

Variables	Medium-Risk		High-Risk	
	aOR (95%CI)	B; *p* Value	aOR (95%CI)	B; *p* Value
Number of responders				
Age groups, years				
25–34	Ref.		Ref.	
35–44	1.66 (1.27–2.18)	0.51; <0.001	1.14 (0.61–2.14)	0.13; 0.68
45–54	2.74 (2.07–3.61)	1.01; <0.001	2.20 (1.19–4.08)	0.79; 0.01
55+	5.99 (4.48–7.99)	1.79; <0.001	6.96 (3.82–12.67)	1.94; <0.001
Sex				
Male	Ref.		Ref.	
Female	0.37 (0.31–0.44)	−1.00; <0.001	0.25 (0.18–0.34)	−1.41; <0.001
Education				
Secondary and lower	Ref.		Ref.	
College	0.99 (0.79–1.25)	−0.01; 0.96	0.84 (0.56–1.26)	−0.18; 0.39
University	0.82 (0.68–0.99)	−0.19; 0.04	0.63 (0.45–0.89)	−0.46; 0.01
Smoking habits				
Never	Ref.		Ref.	
Former	0.98 (0.80–1.20)	−0.02; 0.85	1.32 (0.92–1.89)	0.27; 0.14
Current smokers	1.05 (0.85–1.30)	0.50; 0.65	1.04 (0.70–1.55)	0.04; 0.85
Physical activity in leisure time				
Physically inactive (1st quintile)	Ref.		Ref.	
Physically active (2nd–5th quintile)	0.93 (0.76–1.13)	−0.07; 0.46	0.82 (0.58–1.17)	−0.20; 0.27
Nutrition habits (yes/no)				
More frequent * fast-food consumption	1.04 (0.87–1.24)	0.04; 0.69	1.07 (0.76–1.51)	0.07; 0.70
More frequent consumption of meat products and potatoes	1.41 (1.20–1.65)	0.34; <0.001	1.48 (1.07–2.03)	0.39; 0.02
More frequent fresh vegetables, fruit, and fish consumption	0.83 (0.71–0.97)	−0.19; 0.02	0.67 (0.49–0.90)	−0.41; 0.01
More frequent dairy product consumption	1.07 (0.92–1.25)	0.07; 0.38	1.18 (0.88–1.59)	0.17; 0.27
More frequent sweets consumption	0.75 (0.65–0.88)	−0.28; <0.001	0.45 (0.33–0.61)	−0.80; <0.001
More frequent porridge, cereals, and pasta consumption	0.77 (0.66–0.89)	−0.27; <0.001	0.70 (0.52–0.94)	−0.36; 0.02
Self-rated health				
Good	Ref.		Ref.	
Average + Poor	1.58 (1.34–1.87)	0.46; <0.001	2.41 (1.77–3.28)	0.88; <0.001
***Constant***	-	0.13; 0.52	-	−1.70; <0.001

Adjusted odds ratios (aOR) and 95% confidence intervals (CI) were calculated to determine the independent influence of each variable on the odds of being in a higher-risk profile. More frequent *—the proportions of more-frequent-than-average consumption of food items within each factor-derived food group. B—model coefficient.

**Table 5:** Association of CVD outcome with modifiable biological risk factor profiles, self-rated health, and lifestyle factors.

Variables	Multiple Binary	Logistic Regression
	aOR (95%CI)	B; *p* Value
Number of responders		
Biological risk factors profiles		
Low-risk	Ref.	
Medium-risk	1.59 (1.23–2.06)	0.46; <0.001
High-risk	2.52 (1.70–3.75)	0.93; <0.001
Metabolic syndrome **^#^**		
No	Ref.	
Yes	1.33 (1.06–1.67)	0.29; 0.013
Self-rated health		
Good	Ref.	
Average + Poor	1.41 (1.13–1.77)	0.34; 0.003
Smoking habits		
Never	Ref.	
Former	1.10 (0.83–1.45)	0.1; 0.50
Current smokers	0.86 (0.63–1.16)	−0.15; 0.32
Physical activity in leisure time		
Physically inactive (1st quintile)	Ref.	
Physically active (2nd–5th quintile)	0.95 (0.73–1.26)	−0.05; 0.74
Nutrition habits		
More frequent * fast-food consumption	1.06 (0.82–1.37)	0.06; 0.64
More frequent consumption of meat products and potatoes	1.32 (1.04–1.66)	0.28; 0.02
More frequent fresh vegetables, fruit, and fish consumption	0.99 (0.79–1.22)	−0.02; 0.88
More frequent dairy product consumption	1.01 (0.81–1.25)	0.01; 0.96
More frequent sweets consumption	0.95 (0.76–1.17)	0.06; 0.61
More frequent porridge, cereals, and pasta consumption	0.94 (0.75–1.16)	−0.07; 0.54

More frequent *—The proportions of more-frequent-than-average consumption of food items within each factor-derived food group. Adjusted odds ratios (aOR) and 95% confidence intervals (CI) were calculated to determine the independent influence of each variable on the odds of CVD. The dependent variable was CVD. Independent variables were the three biological risk factor profiles, lifestyle habits (smoking status, physical activity status, nutrition habits), sex, age groups, and education status. ^#^—Metabolic syndrome was included in the model instead of the biological risk factor profiles. B—model coefficient.

## Data Availability

The data used in the present study will not be made publicly available, but they are accessible by contacting the corresponding author.

## Supplementary Materials

The following supporting information can be downloaded at: https://www.mdpi.com/article/10.3390/jcm15093475/s1, Table S1. The distribution of sociodemographic and lifestyle factors according to biological factor profiles. Table S2. The distribution of modifiable biological risk factor profiles, self-rated health, and lifestyle factors according to CVD outcome.

## Abbreviations

The following abbreviations are used in this manuscript:

CVD	Cardiovascular disease
BMI	Body mass index
SBP	Systolic blood pressure
HDL	High-density lipoprotein
LPA	Latent profile analysis
WHO	World Health Organization
SD	Standard deviation
IHD	Ischemic heart disease
AIC	Akaike Information Criterion
BIC	Bayesian Information Criterion
SABIC	Sample-size Adjusted Bayesian Information Criterion
CAIC	Consistent Akaike Information Criterion
aOR	Adjusted odds ratios
Npar	Number of free parameters estimated in the model
LL	Log-likelihood
CI	Confidence interval
