# Supplementary material for: Latent Profiles of Modifiable Biological Factors and Their Associations with Lifestyle Factors and Cardiovascular Disease Outcomes
Source: J Clin Med. 2026 May 1;15(9):3475. doi: 10.3390/jcm15093475 (PMC13163477; doi:10.3390/jcm15093475)
Supplement: Supplementary file 1 [file jcm-15-03475-s001.zip › jcm-4240312-supplementary.pdf]

Table S1. The distribution of sociodemographic and lifestyle factors according to biological factor profiles

Variables	Profiles<br>Low-risk<br>n (%)	 Medium-risk<br>n (%)	 High-risk<br>n (%)	χ^2; p value
Number of responders	1452 (42.6)	1721 (50.4)	238 (7.0)	
Age groups, years,				
25-34	289 (19.9)[a,b]	125 (7.3)[a]	17 (7.1)[b]	349.4; <0.001
35-44	466 (32.1)[a,b]	329 (19.1)[a]	31 (13.0)[b]	
45-54	373 (25.7)	428 (24.9)	49 (20.6)	
55+	324 (22.3)[a,b]	839 (48.7)[a,c]	141 (59.3)[b,c]	
Sex				
Male	455 (31.3)[a]	909 (52.8)[a,b]	151 (63.4)[b]	184.7; <0.001
Female	997 (68.7)[a]	812 (47.2)[a,b]	87 (36.6)[b]	
Education				
Secondary and lower	359 (24.7)[a]	595 (34.6)[a,b]	106 (44.5)[a,b]	78.9; <0.001
College	254 (17.5)	350 (20.4)	47 (19.7)	
University	838 (57.8)a	773 (45.0)[a,b]	85 (35.8)[a,b]	
Smoking habits				
Never	923 (63.6)[a]	972 (56.5)[a,b]	114 (47.9)[a,b]	31.44; <0.001
Former	282 (19.4)[a]	381 (22.2)[b]	71 (29.8)[a,b]	
Current smokers	246 (17.0)[a]	366 (21.3)[a]	53 (22.3)	
Physical activity in leisure time				
Physically inactive (1st quintile)	264 (18.3)	356 (20.8)	58 (24.8)	6.57; 0.038
Physically active (2st - 5st quintile)	1178 (81.7)	1358 (79.2)	176 (75.2)	
Nutrition habits (yes/no)				
More frequent* fast-food consumption	723 (49.9)[a,b]	688 (40.1)[a]	91 (38.4)[b]	33.82; <0.001
More frequent consumption of meat products and potatoes	637 (43.9)[a,b]	1044 (60.8)[a]	156 (65.8)[b]	104.8; <0.001
More frequent fresh vegetables, fruit, and fish consumption	803 (55.4)[a]	885 (51.6)	108 (45.6)[a]	9.88; 0.007
More frequent dairy product consumption	756 (52.1)	915 (53.3)	128 (54.0)	0.576; 0.75
More frequent sweets consumption	836 (57.7)[a,b]	850 (49.5)[a]	87 (36.7)[b]	44.96; <0.001
More frequent porridge, cereals, and pasta consumption	778 (53.7)[a,b]	769 (44.8)[a,c]	100 (42.2)[b,c]	28.53; <0.001
Self-rated health				
Good	1029 (70.9) [a,b]	948 (55.1) [a,c]	103 (43.3) [b,c]	116.147; <0.001
Average+Poor	423 (29.1) [a,b]	773 (44.9) [a,c]	135 (56.7) [b,c]	

More frequent* - the proportion of more-than-average consumption of food items in each factor food group.
[a] - p<0.001 as compared to the Low-risk profile, [b] – p<0.001 as compared to the Medium-risk profile, [c] – p<0.05 as compared to the Medium-risk profile.

Table S2. The distribution of modifiable biological risk factor profiles, self-rated health, and lifestyle factors according to CVD outcome

Variables	CVD No n (%)	CVD Yes n (%)	χ^2; p value
Number of responders	3018 (88.1)	408 (11.9)	
Biological risk factors profiles			
Low-risk	1341 (44.6)	111 (27.5)	56.58; <0.001
Medium-risk	1481 (49.2)	240 (59.4)	
High-risk	185 (6.2)	53 (13.1)	
Metabolic syndrome[#]			
No	2095 (69.7)	229 (56.5)	28.43; <0.001
Yes	911 (30.3)	176 (43.5)	
Self-rated health			
Good	1896 (62.8)	190 (46.6)	39.874; <0.001
Average+Poor	1122 (37.2)	218 (53.4)	
Smoking habits			
Never	1777 (58.8)	242 (59.3)	1.7; 0.44
Former	643 (21.2)	95 (23.3)	
Current smokers	595 (17.0)	71 (17.4)	
Physical activity in leisure time			
Physically inactive (1st quintile)	603 (20.1)	80 (19.8)	0.03; 0.87
Physically active (2st - 5st quintile)	2397 (79.9)	325 (80.2)	
Nutrition habits			
More frequent* fast-food consumption	1363 (45.3)	145 (35.7)	13.2; <0.001
More frequent consumption of meat products and potatoes	1596 (53.0)	250 (61.6)	10.6; 0.001
More frequent fresh vegetables, fruit, and fish consumption	1592 (52.9)	210 (51.7)	0.18; 0.67
More frequent dairy product consumption	1587 (52.7)	219 (53.9)	0.23; 0.64
More frequent sweets consumption	1580 (52.5)	199 (49.0)	1.7; 0.19
More frequent porridge, cereals, and pasta consumption	1470 (48.8)	186 (45.8)	1.28; 0.26

More frequent* - The proportions of more frequent than average consumption of food items in each factor food group.

[#] - Metabolic syndrome was included in the model instead of the biological risk factor profiles.
